# The Nasopharyngeal Microbiome: A Narrative Review of the Hidden Regulator of Ear, Nose, and Throat (ENT) Inflammations

**DOI:** 10.7759/cureus.109921

**Published:** 2026-05-30

**Authors:** Katerina Panagiotidi, Andreas Markidis, Ioannis Karamatzanis, Mohannad Almomani, Rafael Omirou, Panagiota Kosmidou

**Affiliations:** 1 Otolaryngology - Head and Neck Surgery, Mediterranean Hospital of Cyprus, Limassol, CYP; 2 Ear, Nose, and Throat, University of Cambridge, Cambridge, GBR; 3 Internal Medicine, Ipswich Hospital, Ipswich, GBR; 4 Ear, Nose, and Throat, Mediterranean Hospital, Limassol, CYP; 5 Nursing, Cyprus University of Technology (CUT), Limassol, CYP; 6 Nursing, Mediterranean Hospital of Cyprus (MHOC), Limassol, CYP; 7 Otolaryngology - Head and Neck Surgery, University of Patras, Medical School, Patras, GRC

**Keywords:** artificial intelligence in medicine, bacterial dysbiosis, chronic otitis media, chronic rhinosinusitis, commensal microbial flora, dolosigranulum pigrum, ent inflammatory diseases, microbial homeostasis, nasopharyngeal microbiome, precision – medicine

## Abstract

The nasopharyngeal microbiome is a central regulator of respiratory health. The upper airway microbial community acts as the primary gatekeeper against respiratory pathogens and maintains homeostasis in the upper respiratory tract (URT). This community is established at birth and influenced by the delivery method and antibiotic exposure. Disruptions to this balance are recognised as a major driver of chronic inflammatory ear, nose, and throat (ENT) diseases. This review analyses the literature on the relationship between the nasopharyngeal microbiome and inflammatory ENT diseases. We searched recent literature (2015-2025) via PubMed and Scopus, focusing on 16S rRNA and metagenomic studies of the upper respiratory tract. We examined papers that linked microbial shifts to clinical outcomes in otitis media, rhinosinusitis, and allergic rhinitis, as well as studies applying machine learning to diagnostic modelling. Clinical health is associated with stable colonisation by *Dolosigranulum* and *Corynebacterium*. These commensals protect the host by maintaining the mucosal barrier and competing against pathogens. Chronic disease, in contrast, is marked by a bloom of *Streptococcus*, *Haemophilus*, or *Moraxella*. In chronic rhinosinusitis, loss of bacterial diversity and *S. aureus *biofilm formation often lead to treatment failure. Machine learning tools like Random Forest and XGBoost classifiers have been applied to nasopharyngeal microbiome data. In published cohorts, these models have achieved sensitivity and specificity values of 80-90% for identifying dysbiotic profiles associated with disease, outperforming standard culture in speed and taxonomic resolution. These findings support a shift from broad antibiotic use toward microbiome-informed treatment. Standardising sampling and sequencing methods remains the next necessary step.

## Introduction and background

Microbial communities have a fundamental role in shaping health and disease [1]. The nasopharyngeal microbiome, though less studied than that of the gut, is a central component of upper respiratory tract defence.

The nasopharyngeal microbiome develops from the first days of life. Mode of delivery, diet, antibiotic use, environment, and exposure to infectious agents all shape its composition [2,3]. Key genera include *Streptococcus, Moraxella, Haemophilus, Corynebacterium*, and *Dolosigranulum,* which maintain a balance between symbiosis and pathogenicity [4,5].

These microorganisms colonise mucosal surfaces, regulate local and systemic immune responses, inhibit pathogen adhesion, and produce protective metabolites. A healthy microbial flora in the nasopharynx is associated with reduced rates of otitis media, chronic rhinosinusitis, pharyngitis, and allergic rhinitis [4,6,7].

Microbial imbalance (dysbiosis) is linked to both acute and chronic disease, affecting treatment response [8-10]. Despite growing interest, the specific mechanisms by which dysbiosis triggers ear, nose, and throat (ENT) disease remain poorly defined. Longitudinal data linking microbial shifts to clinical onset are still limited. This review addresses that gap by synthesising current evidence on the nasopharyngeal microbiome in ENT inflammatory disease and examining the emerging role of artificial intelligence in data analysis.

## Review

Literature search strategy

A structured narrative search was conducted in PubMed, Scopus, and Google Scholar to identify peer-reviewed articles published between 2015 and 2025. Key terms included "nasopharyngeal microbiome," "dysbiosis," "otitis media," "chronic rhinosinusitis," "artificial intelligence," and "machine learning." Studies were selected based on relevance to the nasopharyngeal microbial ecosystem and the application of computational models in ENT diagnostics. Longitudinal cohort studies, meta-analyses, and high-impact reviews using 16S rRNA or metagenomic sequencing were prioritised. 

Composition and function of the nasopharyngeal microbiome

**Figure 1:**
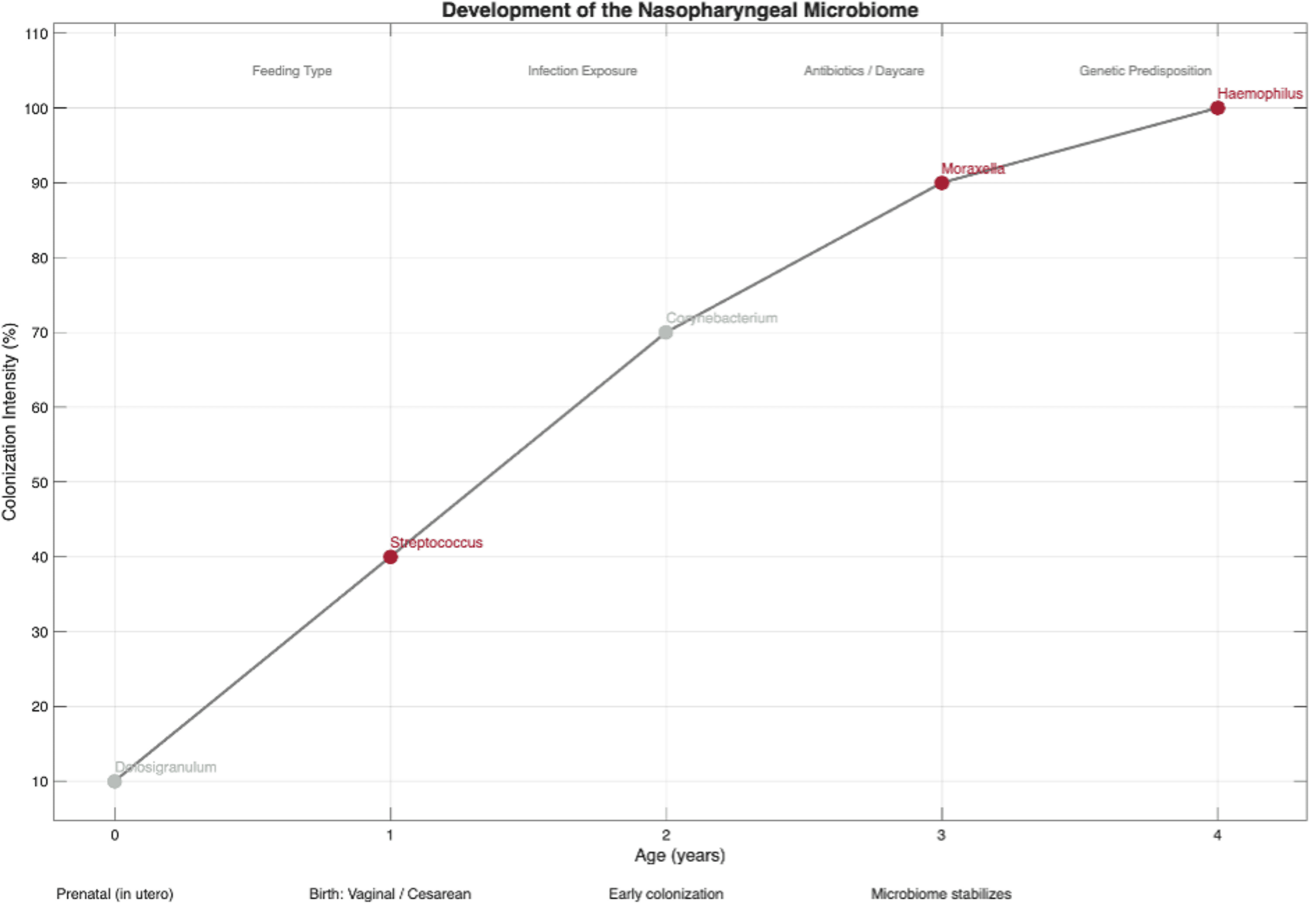
Development of the Nasopharyngeal Microbiome The graph illustrates the successional intensity of microbial colonisation from the prenatal period to age four. The key clinical point from this graph is the early dominance of Dolosigranulum in the first months of life, followed by a shift toward Haemophilus and Moraxella by ages three to four. This transition coincides with daycare exposure and antibiotic courses. The graph supports the hypothesis that early microbial succession patterns predict later respiratory health outcome [1,2,11,13].

The nasopharyngeal microbiome is a complex microbial ecosystem present from early life, as illustrated in Figure 1. Whether colonisation truly begins in utero remains debated. Some studies report bacterial DNA in amniotic fluid and meconium, but contamination cannot be excluded in most [3,11]. What is better established is that colonisation intensifies during birth, particularly with vaginal delivery, and stabilises within the first years of life [3,11]. Composition is shaped by feeding type, infection exposure, antibiotic use, daycare attendance, and genetic predisposition [6,7,11]. 

Under normal conditions, dominant genera include *Dolosigranulum, Corynebacterium, Streptococcus, Moraxella*, and *Haemophilus *[7,12]. The first two have a mainly protective role, strengthening the mucosal barrier and modulating inflammatory responses. Their presence is associated with fewer upper respiratory infections [4,6,13].

Elevated levels of *Streptococcus pneumoniae, Haemophilus influenzae, and Moraxella catarrhalis* are associated with greater susceptibility to otitis media, pharyngitis, and sinusitis [7,10,13]. However, carriage of these organisms does not inevitably cause disease. Pathogenicity depends on the balance among the wider microbial community, host immune status, and concurrent viral infection, not on the presence of any single taxon. 

The microbiome also regulates local immunity. Some bacteria stimulate mucosal IgA production or regulatory T cells, while others induce cytokines such as interleukin (IL)-8 and tumor necrosis factor (TNF)-α [7,14,15]. Reduced microbial diversity has been correlated with impaired antimicrobial defence and increased vulnerability to chronic inflammation [10,12].

The use of 16S rRNA sequencing and shotgun metagenomics now enables precise identification of microbial species and metabolic pathways, thereby defining clinically relevant microbial profiles [7,10,16,17].

ENT inflammatory diseases

Otitis Media

Otitis media, especially acute otitis media (AOM) and chronic otitis media effusion (COME), is one of the most common childhood infections. While traditional interpretations focused on pathogens such as *S. pneumoniae, H. influenzae, *and *M. catarrhalis*, current evidence highlights the regulatory role of the nasopharyngeal microbiome. However, the modern approach recognises the importance of the microbial ecology of the nasopharynx as a central regulatory factor [3,6,7,13]. 

Studies using 16S rRNA sequencing have shown that children with COME exhibit a significantly altered nasopharyngeal microbiome compared to healthy children. Specifically, a reduction in beneficial genera such as *Corynebacterium* and *Dolosigranulum* is observed, alongside an increased abundance of pathogenic taxa such as *Haemophilus* and *Streptococcus *[6,18]. This dysbiosis is considered a key factor that facilitates the establishment and development of middle ear infections.

In the study by Laufer et al. [13], the alteration of microbiome balance preceded the onset of otitis media, supporting the idea that changes in the microbial community may have predictive value. Moreover, the study by Jörissen et al. [19] in children with otitis media with effusion demonstrated an increased presence of *Streptococcus* in the nasopharynx, while a healthy microbiome was associated with higher levels of *Dolosigranulum pigrum*, a bacterium with antimicrobial activity through the production of hydrogen peroxide.

Another important factor that appears to influence the composition of the microbiome is the use of antibiotics, which may increase the predisposition to recurrent episodes of otitis media. The study by Liu et al. [16] in children with recurrent otitis media demonstrated significantly lower microbial diversity and a reduction in protective strains.

Similar dysbiotic patterns are seen further along the upper airway, with the sinus microbiome showing comparable shifts in chronic disease.

Rhinosinusitis

Rhinosinusitis, whether acute or chronic, is one of the most common inflammatory conditions of the upper respiratory tract and is closely linked to the structure and lifelong evolution of the nasopharyngeal microbiome. Recent progress in nasal and sinus microbiology has revealed that microbial colonization acts as a key regulator of the inflammatory response rather than a passive factor. 

The nasal cavity and paranasal sinuses harbor a rich and stable microbiome, mainly composed of *Corynebacterium, Dolosigranulum, Staphylococcus, and Moraxella* [7]. Under normal conditions, these microbes form an ecological barrier against pathogen colonization. In rhinosinusitis, especially in chronic cases, this balance is disturbed, leading to dysbiosis [10].

Studies have shown that patients with Chronic Rhinosinusitis (CRS) exhibit reduced microbial diversity and a dominance of potentially pathogenic species, including *Staphylococcus aureus, Pseudomonas aeruginosa, and Haemophilus influenzae*. Notably, the presence of *Corynebacterium accolens* is associated with better clinical outcomes, while its absence correlates with more severe inflammation [10,20].

According to Hoggard et al. [10], metagenomic analysis also revealed changes in the mycobiome, which further aggravate inflammation. In CRS with nasal polyposis [as defined by European Position Paper on Rhinosinusitis and Nasal Polyps (EPOS) guidelines 2020], *Aspergillus* and *Candida *species were more abundant, whereas *Malassezia* and *Penicillium* predominated in CRS patients without polyps.

In addition, the meta-analysis by Wagner Mackenzie et al. [21] suggests that persistent inflammation may result not from individual pathogens but from disruption of the entire microbial community. The stability of this community, its antimicrobial activity, and its role in mucosal immune regulation appear equally important.

Thus, rhinosinusitis represents an inflammatory model in which the microbiome functions as an active modulator of disease. This understanding supports the development of personalized therapeutic approaches based on the patient’s specific nasopharyngeal microbiome profile.

The pharynx is similarly affected by microbial community disruption, where dysbiosis has been linked to both acute and recurrent disease.

Pharyngitis

Acute pharyngitis is one of the most common reasons for visits to ENT clinics or even emergency departments, in both pediatric and adult populations. Despite the traditional distinction between viral and bacterial causes, modern microbiological approaches examine the role of the overall microbial community of the nasopharynx as a potential regulator of the inflammatory response in pharyngeal infections.

 Beneficial bacteria, such as *Dolosigranulum pigrum* and *Corynebacterium accolens*, can compete almost equally with pathogenic strains like *Streptococcus pyogenes,* reducing the likelihood of streptococcal pharyngitis [4,22]. Especially in children, microbial community homeostasis is associated with fewer pharyngitis episodes and reduced indications for antibiotic therapy [3,12,13].

In the study by Man et al. [7], individuals with chronic or recurrent pharyngitis had different microbial profiles compared to healthy subjects, with a higher presence of *Streptococcus anginosus *and *Prevotella *spp., correlating with increased inflammatory response. Additionally, the possible presence of upper respiratory viruses (e.g., rhinoviruses, adenoviruses) combined with microbial dysbiosis creates a “pro-inflammatory microenvironment” that exacerbates symptoms and prolongs resolution.

Integrating microbiome data into pharyngitis diagnosis could improve differential diagnosis and reduce irrational antibiotic use. Microbial profile analysis may, in the future, serve as a prognostic tool for chronic or recurrent pharyngitis, especially in vulnerable populations such as children, immunocompromised patients, and the elderly.

Allergic Rhinitis

Allergic rhinitis (AR) is a chronic inflammatory disorder of the nasal mucosa, triggered by allergens and primarily characterized by nasal congestion, sneezing, rhinorrhea, and itching. While its pathophysiology is well established regarding type I (IgE-mediated) immune responses, recent interest has focused on the role of the nasopharyngeal microbiome in disease expression and regulation, forming the basis for therapies involving biological agents.

Studies have shown that patients with allergic rhinitis exhibit significant differences in their microbial profiles compared to healthy individuals. Specifically, Mahdavinia et al. [23] found reduced diversity in the nasal microbiome of AR patients, dominated by *Staphylococcus* and *Corynebacterium* strains, with relatively lower levels of “protective” bacteria such as *Dolosigranulum*.

This dysbiosis may affect local immune regulation by disrupting epithelial cell function and endogenous antimicrobial peptides. Moreover, disrupted microbial balance appears linked to increased production of cytokines IL-4, IL-5, and IL-13, which enhance Th2 responses and eosinophilic inflammation [15].

Interestingly, it has been hypothesized that allergic rhinitis may partly result from early-life microbiome alterations. Bosch et al. [11] reported that children born by cesarean section or fed formula had lower respiratory microbiome diversity and higher rates of AR in preschool age.

Lastly, research is exploring microbiome-targeted interventions. Nasal probiotic or postbiotic formulations, such as those based on *Lactobacillus sakei *or *Dolosigranulum pigrum,* are being studied for their potential to restore homeostasis and reduce allergen sensitivity [6,15,24].

Viral Upper Respiratory Infections

Viral infections of the upper respiratory tract, including the common cold, influenza, and respiratory syncytial virus (RSV), remain leading causes of morbidity worldwide, particularly among infants, children, and the elderly. The nasopharyngeal microbiome appears to play an important role in susceptibility, immunity, and disease severity.

Studies indicate that microbiome composition can either predispose to or protect against viral pathogens. The presence of *Dolosigranulum pigrum* and *Corynebacterium accolens *has been linked to reduced RSV severity, likely through maintenance of epithelial integrity and modulation of local immune responses [16]. In contrast, overgrowth of *Moraxella* or *Haemophilus* in infants is associated with more severe and prolonged infections, possibly due to increased mucosal permeability and viral penetration into the underlying epithelium [25,26].

Willie et al. found that *Streptococcus pneumoniae* detected in sputum samples in children correlates with higher upper respiratory tract infection (URTI) risk, supporting the notion that normal microbial colonization has both protective and regulatory roles [27]. Microbial shifts can emerge early during infection and may serve as prognostic indicators of severity.

Incorporating microbial profiles into predictive models could improve early identification of severe URTIs, particularly in neonates and high-risk groups. Moreover, restoring microbial balance post-infection may reduce illness duration and prevent secondary bacterial complications. These insights are especially relevant in the COVID-19 era, where notable changes in the nasopharyngeal microbiome of SARS-CoV-2 patients further highlight the complex microbiome-virus-host interplay.

Microbiome and ENT Inflammatory Diseases

**Figure 2:**
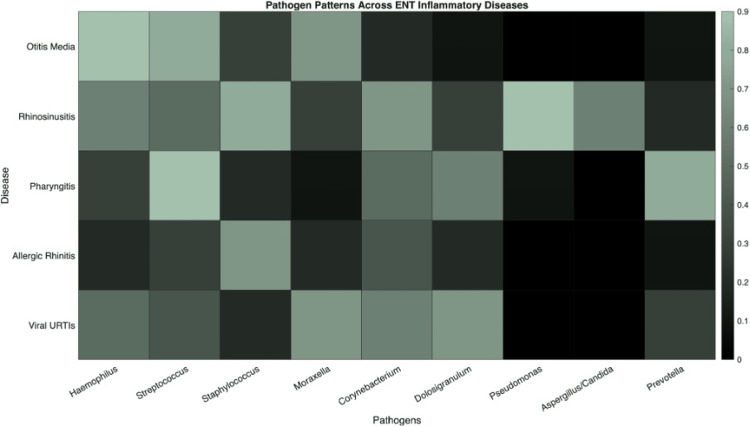
Pathogen Patterns Across ENT Inflammatory Diseases Heatmap of the relative dominance of microbial taxa across the ENT diseases discussed in this review [3,4,6,8,9,15,16]. The associations shown are based on observational and sequencing data, and they are associative, not causal. No formal statistical framework underpins the heatmap directly. It is a visual synthesis of findings across heterogeneous studies. It should be interpreted with this limitation in mind. Each row= ENT disease; Each column= pathogen; Blue= more dominant/associated; Black= low/absent.

The distribution and dominance of specific microbial taxa vary significantly across different ENT pathological states. Figure 2 summarizes the characteristic pathogen patterns associated with the major inflammatory conditions discussed in this review.

Discussion

The evidence reviewed here consistently shows that the nasopharyngeal microbiome is an active participant in ENT inflammatory disease, not a passive bystander. Across otitis media, rhinosinusitis, pharyngitis, allergic rhinitis, and viral URTIs, the same pattern emerges: depletion of protective commensals and overgrowth of opportunistic taxa precede or accompany disease. This shared mechanism suggests that microbiome-based diagnostics could cut across multiple ENT conditions rather than being disease-specific.

The Role of Artificial Intelligence in ENT Practice

Artificial intelligence (AI) and machine learning (ML) are being increasingly applied to complex microbiome datasets, with growing potential for clinical use in ENT. Their application to nasopharyngeal microbiome data is still early-stage, but several findings are promising.

AI-driven profiling from routine nasopharyngeal swabs could enable real-time diagnostics. Published studies have applied tools including Random Forest, XGBoost, and support vector machine (SVM) classifiers to 16S rRNA sequencing data. In these cohorts, models have achieved sensitivity and specificity in the range of 80-90% for identifying dysbiotic profiles linked to conditions such as chronic rhinosinusitis and otitis media [28,29]. This compares favourably with conventional culture, which misses fastidious organisms and returns results in 48-72 hours.

It is important to note that AI-based microbiome profiling should currently be regarded as a preliminary diagnostic aid, not a replacement for standard microbiological testing. A positive AI-predicted dysbiosis signal should prompt, rather than replace, confirmatory laboratory investigation. Over-reliance on AI output without microbiological confirmation risks misdiagnosis and inappropriate antibiotic prescribing. The practical model that best fits current evidence is AI as a triage tool: flagging high-risk patients for expedited culture and targeted workup.

Machine learning trained on large multi-omics cohorts may also predict disease progression or therapeutic response based on individual microbial signatures [29]. These capabilities could support targeted antibiotic selection, probiotic therapy, or microbiome-modulating interventions optimised through AI-assisted feature selection [30].

Beyond diagnostics, AI is being used in systems biology to design microbiome-based therapeutics and vaccines by modelling host-microbe interactions [31,32]. Large initiatives such as the Human Microbiome Project and platforms like GMrepo already use machine learning to identify microbial disease signatures [33,34].

Significant challenges remain. Microbiome pipelines are not yet standardised, limiting comparability across studies. Many AI models are difficult to interpret clinically. Ethical deployment in routine care requires further work [34,35]. Nonetheless, AI has real potential to improve diagnostic precision, refine treatment strategies, and advance the management of ENT airway inflammation.

## Conclusions

The nasopharyngeal microbiome plays a central and active role in shaping inflammatory responses in upper airway disease. Evidence highlights its diversity, functional importance, and sensitivity to environmental and therapeutic influences. The microbial shifts associated with otitis media, chronic rhinosinusitis, pharyngitis, allergic rhinitis, and viral URTIs are now well enough characterised to serve as diagnostic and prognostic markers.

Applying AI to microbiome data offers a practical route to translate these findings into clinical care, improving diagnostics, predicting outcomes, and tailoring treatment. Future work should standardise data collection and sequencing methods, integrate clinical and microbial datasets, and build databases to support predictive, preventive, and personalised ENT medicine. In practice, standardisation means agreeing on swab site, DNA extraction protocols, sequencing depth, and bioinformatic pipelines across centres. Without this, AI models trained on one dataset will not generalise to another.
